# Temperature-Dependent Mechanical Properties of Graphene/Cu Nanocomposites with In-Plane Negative Poisson's Ratios

**DOI:** 10.34133/2020/5618021

**Published:** 2020-02-05

**Authors:** Yin Fan, Yang Xiang, Hui-Shen Shen

**Affiliations:** ^1^School of Engineering, Western Sydney University, Locked Bag 1797, Penrith, NSW 2751, Australia; ^2^School of Aeronautics and Astronautics, Shanghai Jiao Tong University, Shanghai 200240, China; ^3^School of Ocean and Civil Engineering, Shanghai Jiao Tong University, Shanghai 200240, China

## Abstract

Negative Poisson's ratio (NPR), also known as “auxetic”, is a highly desired property in a wide range of future industry applications. By employing molecular dynamics (MD) simulation, metal matrix nanocomposites reinforced by graphene sheets are studied in this paper. In the simulation, single crystal copper with crystal orientation [1 1 0] is selected as the matrix and an embedded-atom method (EAM) potential is used to describe the interaction of copper atoms. An aligned graphene sheet is selected as reinforcement, and a hybrid potential, namely, the Erhart-Albe potential, is used for the interaction between a pair of carbon atoms. The interaction between the carbon atom and copper atom is approximated by the Lennard-Jones (L-J) potential. The simulation results showed that both graphene and copper matrix possess in-plane NPRs. The temperature-dependent mechanical properties of graphene/copper nanocomposites with in-plane NPRs are obtained for the first time.

## 1. Introduction

Graphene is an ideal reinforcement agent for high-performance composites due to its superb mechanical and physicochemical properties [1–6]. Recently, polymer- and metal-based composites [7–21] reinforced by graphene have gained tremendous attention from the scientific community. Compared with polymer-based composites, composites with metal matrix can provide higher stiffness and strength and service under a higher temperature, which makes them have the potential for more critical applications in industry, especially in the aerospace and automobile industry. Several researchers have reported in recent years [9–21] on experimental and numerical studies of graphene-reinforced metal composites. The numerical studies [16–21] mainly based on the method of molecular dynamics (MD) simulations indicated that the material properties of metal composites can be significantly improved by applying single-layer graphene reinforcement. In most experimental reports, the graphene used to reinforce metal matrix composites (MMCs) is mainly multilayered graphene (MLG) or graphene nanoplatelets (GNPs) which contain 10-100 layers of carbon atoms. It is noted that the material properties of these multilayer graphene derivatives are far less superior to those of the single-layer graphene counterpart [14]. It was also observed [15] that increasing the number of graphene layers may degrade the mechanical and other properties of these MMCs.

Negative Poisson's ratio (NPR), also known as “auxetic”, is a phenomenon associated with a material that experiences transverse expansion when a tensile load is applied in the longitudinal direction. On the other hand, if the material is under a compressive load, it contracts transversely. The NPR materials are a novel class of materials, which have advantages that conventional materials may not possess [22]. Materials with NPR have triggered significant interest in the scientific community because of their unique physical properties [23–27] including high shear modulus, high indentation resistance [28], large plane strain fracture toughness [29], and excellent shock and sound absorption abilities [30, 31], and all of which are highly desirable properties for engineering applications. Specifically, the NPR materials can be used in microelectromechanical systems (MEMS) and fastening devices due to their auxetic characteristics, and they can be also added into engineering structures (e.g., sandwich or laminated panels in the aerospace industry) as energy absorption components. The natural materials found with NPR are polymers with microporous characteristics. Such polymer consists of a randomly or periodically distributed 3D array of particles interconnected by fibrils in the microscopic scale. For instance, microporous polytetrafluoroethylene (PTFE) film has a network of individual fibrils which can be extended by several microns in both directions when under uniaxial tension [32, 33]. On the other hand, auxetic materials could be achieved via designing and tailoring their microscopic compositions and structures. Such man-made materials consist of a large number of unit cells, which are arranged into certain periodical truss patterns such as reentrant type, chiral type, or rotating units [32]. Recently, it has been reported that laminated composites reinforced with microfibers can gain out-of-plane NPR [34] through design and optimization of stacking sequences. However, there are still some limitations in engineering applications for the above NPR materials. For example, the majority of materials fabricated only have NPR in certain directions where the stiffness is low in those directions. Hence, such materials may only be used as the core for sandwiches which need to be enhanced by composite face sheets. In addition, NPR is usually achieved in the out-of-plane direction of a structural element rather than in the in-plane direction [35–37]. The research on composites with in-plane NPR is still in the conceptual stage [38, 39] due to the unavailability of such type of NPR materials and their associated material properties for structural applications. Nevertheless, as hinted in the literature [40], there is a possible approach to obtain in-plane auxetic composites by using the matrix and the reinforcing phase that both possess the in-plane auxetic property.

In most polycrystalline metal materials, the value of Poisson's ratio is in the vicinity of 1/3. However, it is different for metal materials with single crystals where NPR may exist in certain crystallographic directions [41]. Early research [42] suggested that some single crystal metals including copper with a face-centered cubic (FCC) lattice might have an auxetic phenomenon in the $\left[1\;\bar{1}\;0\right]$ direction when subjected to uniaxial [1 1 0] loading. To date, the NPR for single-layer graphene has been observed by researchers using different approaches, including the Monte Carlo method [43], molecular dynamics and statics [44, 45], molecular structural mechanics [46], first principle theory [47], and membrane theory [48].

In this study, the classical MD method using the LAMMPS package [49] is employed to construct the single crystal copper and the software package OVITO [50] is utilized to generate graphic presentations from the MD simulation results. For graphene, discrepancies exist in the MD studies on Poisson's ratio due to the selection of different potentials for the C-C bond. From existing literature [51], the absolute NPR (-0.158) can be obtained by MD simulations with the Tersoff potential [52] but cannot be achieved with the potentials based on a reactive empirical bond order (REBO) potential [53]. Hence, a hybrid potential [54] based on both REBO and Tersoff potentials is used to address this issue. Finally, the MD models of graphene-reinforced copper composites are established and used for the prediction of the temperature-dependent NPRs and other material properties for the composites.

## 2. MD Models

### 2.1. Single Crystal Copper

**Figure 1:**
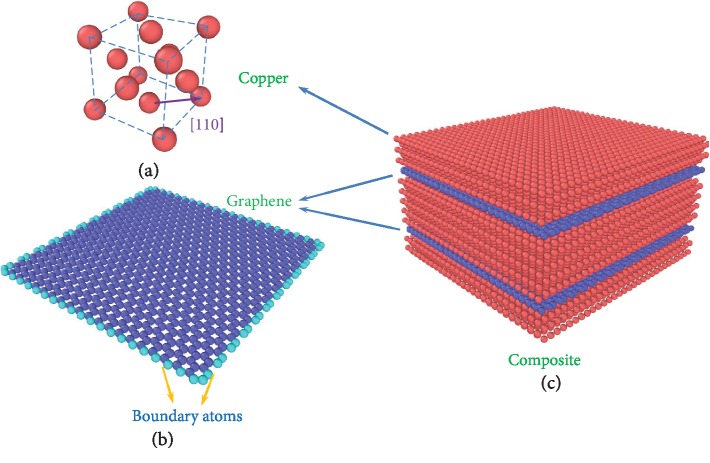
The schematic diagrams of the (a) single crystal copper cell, (b) single layer graphene, and (c) graphene-reinforced copper composite.

Copper is an ideal substrate that supports growing graphene layers. In this study, we create a MD model of single crystal copper along the [1 1 0], $\left[1\;\bar{1}\;0\right]$, and [0 0 1] crystal orientations as shown in Figure 1(a). The simulation box containing 5632 copper atoms is of 40.89 Å × 40.89 Å × 39.76 Å in dimensions. The boundary conditions are set as periodical in the three directions. An embedded-atom method (EAM) potential [55] developed by Dawn and Baskes [56] is employed to create the interaction between each pair of two copper atoms. The mathematical form of the general total energy for a pair of copper atoms is

(1) $$\begin{array}{c} E_{\text{tot}}=\underset{i}{\sum }F_{i}\left[\underset{j\neq i}{\sum }\rho_{j}^{a}\left(R_{ij}\right)\right]+\frac{1}{2}\underset{i}{\sum }\underset{j\neq i}{\sum }\phi_{ij}\left(R_{ij}\right), \end{array}$$

where *F*_*i*_(*ρ*) is the embedding energy of atom *i* which is a function of the atomic electron density *ρ*, *ϕ* is the pair potential interaction, and *α* and *β* are the element types of atoms I and J. In the case of this study, *α* and *β* are the same. The lattice constant and the atomic weight of the FCC copper are selected as 3.615 Å and 63.55, respectively.

In the simulation, the tensile and shear behaviors controlled by strain are applied to this MD model to study its in-plane and out-of-plane mechanical properties. The temperature varying from 300 K to 1000 K is also considered in the simulation to observe the effect of temperature on the mechanical properties of the single crystal copper.

### 2.2. Single-Layer Graphene

Graphene is a two-dimensional material. However, we cannot ignore its effective thickness in the mechanical analysis of Young's modulus and shear modulus. The MD model of the monolayer graphene with 201.68 Å × 200.21 Å is built, which is, respectively, referred to as the zig-zag direction and the armchair direction. A hybrid empirical bond order potential [54] for the C-C interaction based on REBO [53] and Tersoff [52] potentials is employed herein. The cohesive energy of this potential is written as a sum over individual bond energies:

(2) $$\begin{array}{c} E_{\text{C}}=\underset{i>j}{\sum }f_{\text{C}}\left(r_{ij}\right)\left[V_{R}\left(r_{ij}\right)-\frac{b_{ij}+b_{ji}}{2}V_{\Lambda }\left(r_{ij}\right)\right], \end{array}$$

where *V*_*R*_ and *V*_*Λ*_ are the pairwise attractive and repulsive contributions, respectively, *f*_C_ is the cutoff function, *b* is the bond order, and *r* is the distance between the nearest two carbon atoms. The detailed definitions of these physical quantities can be found in the literature [54]. The C-C bond length is well known as 1.42 Å, and the atom weight is 12.011. For the sake of convenience, this hybrid potential is named as the Erhart and Albe potential (EAP).

To determine the in-plane properties, such as *E*_11_, *E*_22_, *G*_12_, *ν*_12_, and *ν*_21_, the simulation tests in three directions are carried out. The force *f* is only applied on the carbon atoms closest to the boundary for each simulation, as shown in Figure 1(b). If we assume that there are *M* atoms in the zigzag direction and *N* atoms in the armchair direction, then we can first obtain stretching rigidities (*C*_11_ and *C*_22_) and shear rigidity (*C*_66_) as:

(3) $$\begin{array}{c} C_{11}=E_{11}h=\frac{{MfL}_{x}}{L_{y}\Delta L_{x}}, \end{array}$$

(4) $$\begin{array}{c} C_{22}=E_{22}h=\frac{{NfL}_{y}}{L_{x}\Delta L_{y}}, \end{array}$$

(5) $$\begin{array}{c} C_{66}=G_{12}h=\frac{{MfL}_{y}}{L_{x}\Delta L_{x}} \end{array}$$

or

(6) $$\begin{array}{c} \;C_{66}=G_{12}h=\frac{{NfL}_{x}}{L_{y}\Delta L_{y}}, \end{array}$$

where *L*_*x*_ and *L*_*y*_ are the origin lengths of the graphene in the zig-zag direction and the armchair direction, respectively, and Δ denotes the deformation caused by the force. It is worth noting that Equations (5) and (6) are valid when Δ*L*_*x*_ or Δ*L*_*y*_ is very small.

There is divergence on the effective thickness of graphene which affects the values of its moduli. Usually, the graphite thickness is used to estimate the effective thickness of graphene. However, this method is not accurate for a mechanical concept. In this study, we treat the graphene sheet as a thin plate in the framework of mechanics and the effective thickness of the graphene sheet will be obtained after the central deflection of graphene is measured under a uniform load. Shen [57] has derived the relationship between the load *q* and the central deflection $\bar{W}$ based on the classical plate theory:

(7) $$\begin{array}{c} \frac{{qL}_{x}^{4}}{D_{11}h_{\text{e}}}=A_{W}^{\left(1\right)}\left(\frac{\bar{W}}{h_{\text{e}}}\right)+A_{W}^{\left(3\right)}{\left(\frac{\bar{W}}{h_{\text{e}}}\right)}^{3}+\cdots , \end{array}$$

where *D*_11_ is the flexural rigidity of graphene in the zig-zag direction. *A*_*W*_^(1)^ and *A*_*W*_^(3)^ are parameters and defined as

(8) $$\begin{array}{c} A_{W}^{\left(1\right)}=\frac{\pi^{6}}{16}\left(1+2c_{4}\beta^{2}+c_{3}\beta^{4}\right), \end{array}$$

(9) $$\begin{array}{c} A_{W}^{\left(3\right)}=\frac{3\pi^{6}}{64}\left[\left(\frac{1}{g_{13}}+\frac{c_{3}\beta^{4}}{g_{31}}\right)\left(1+2c_{4}\beta^{2}+c_{3}\beta^{4}\right)+\left(1+c_{3}\beta^{4}\right)\right]\left(1-\nu_{12}^{\text{G}}\nu_{21}^{\text{G}}\right), \end{array}$$

(10) $$\begin{array}{c} g_{13}=1+18c_{4}\beta^{2}+81c_{3}\beta^{2}, \end{array}$$

(11) $$\begin{array}{c} g_{31}=81+18c_{4}\beta^{2}+c_{3}\beta^{4}, \end{array}$$

(12) $$\begin{array}{c} \beta =\frac{L_{x}}{L_{y}}, \end{array}$$

(13) $$\begin{array}{c} c_{3}=\frac{C_{22}}{C_{11}}, \end{array}$$

(14) $$\begin{array}{c} c_{4}=\nu_{21}^{\text{G}}+2\left(\frac{C_{66}}{C_{11}}\right)\left(1-\nu_{12}^{\text{G}}\nu_{21}^{\text{G}}\right), \end{array}$$

where superscript *G* represents graphene. Finally, the effective thickness of the graphene sheet (*h*_e_) can be derived from Equation (7):

(15) $$\begin{array}{c} h_{\text{e}}={\left[\frac{\left(12\left(1-\nu_{12}^{\text{G}}\nu_{21}^{\text{G}}\right){qL}_{x}^{4}/C_{11}\right)-A_{W}^{\left(3\right)}{\bar{W}}^{3}}{A_{W}^{\left(1\right)}\bar{W}}\right]}^{1/2}. \end{array}$$

The detailed derivation for the load-deflection relationship in Equation (7) is presented in the appendix. In the simulation, the boundary atoms are fixed and a small transverse force is applied to the rest of the atoms.

Once the effective thickness is determined by MD simulations, the effective Young's modulus and shear modulus of the graphene sheet can be obtained uniquely from Equations (3) to (6). The TECs at different temperatures can also be obtained from the relaxation process. For the computation of the TEC, the standard relationship is used [58, 59]:

(16) $$\begin{array}{c} \alpha_{11}=\frac{1}{L_{x0}}\frac{\Delta L_{x}}{\Delta T}=\frac{1}{L_{0}}\frac{L_{x}-L_{x0}}{T-T_{0}}, \end{array}$$

(17) $$\begin{array}{c} \alpha_{22}=\frac{1}{L_{y0}}\frac{\Delta L_{y}}{\Delta T}=\frac{1}{L_{0}}\frac{L_{y}-L_{y0}}{T-T_{0}}, \end{array}$$

where *α* is the TEC with unit K^−1^. *T* and *T*_0_ are the simulation temperature and reference temperature, respectively. *L*_*x*0_ and *L*_*y*0_ are the lengths of graphene in the zig-zag direction and the armchair direction, respectively, corresponding to the reference temperature *T*_0_.

### 2.3. Graphene-Reinforced Copper Nanocomposite

The MD model of the graphene-reinforced copper nanocomposite is illustrated in Figure 1(c). The EAM and EAP potentials are also used in the simulation of composite materials for the interactions of Cu-Cu and C-C. The Lennard-Jones (L-J) potential which is theoretically a good molecular dynamics model for long and short distances for neutral atoms and molecule is adapted for the C-Cu interaction. Moreover, the C-Cu interaction has already been demonstrated to meet the prediction from the Lennard-Jones potential in previous investigations [60, 61]. This potential has also been widely used in existing literature [62–64] on the MD study of carbon/copper molecular structures. To estimate the interaction between copper and carbon atoms, the L-J potential is used:

(18) $$\begin{array}{c} E_{ij}=4\varepsilon \left[{\left(\frac{\sigma }{r}\right)}^{12}-{\left(\frac{\sigma }{r}\right)}^{6}\right]. \end{array}$$

In Equation (18), *r* is the distance between two atoms. Obviously, this potential depends on the depth *ε* and the equilibrium interatomic distance *σ*. These parameters are determined to be *ε* = 0.01996 eV and *σ* = 3.225 Å based on existing studies [65]. On the other hand, the common divisor of the two atoms' lattice parameters is considered in the geometric design of the MD models for the composites to reduce the effect of mismatch, which will lead to internal stress in the MD models during the phases of relaxation and loading.

In the simulation, five different weight fractions (2.8%, 3.7%, 4.8%, 6.1%, and 7.1%) of the graphene sheet are taken into account. The temperature effect is also considered in the simulation. The time step is set as 1 fs, and the thermal relaxation consumes 40000 steps within the context of an isothermal-isobaric (NPT) ensemble. The tensile deformation of the simulation box is controlled by a strain rate of 10^−6^/fs, and the whole load process takes 100000 steps while it takes only 10000 steps for the shear deformation.

## 3. Results and Discussion

### 3.1. Mechanical Properties and NPR of Single Crystal Copper

As a typical FCC metal, single crystal copper shows an in-plane auxetic property when the load is applied in the [1 1 0] direction.  Table 1 shows the in-plane properties of the single crystal copper. From the table, in-plane NPRs (*ν*_12_ and *ν*_21_) are obtained through linear fitting MD simulation results and it is found that auxeticity is more significant with the increase in temperature. However, the elastic moduli (*E*_11_ and *E*_22_) and shear modulus (*G*_12_) are decreased as temperature rises. We also find that the shear modulus is much smaller than the elastic moduli. The thermal expansion coefficient is also obtained and listed in Table 1. The out-of-plane properties of the single crystal copper, including *E*_33_, *G*_13_, and *G*_23_, are listed in Table 2. It is found that the out-of-plane elastic modulus is nearly half of the in-plane elastic modulus. The out-of-plane shear moduli *G*_13_ and *G*_23_ are almost four times of the in-plane shear modulus.

### 3.2. In-Plane Properties and Out-Of-Plane Bending Behavior of the Graphene Sheet

The variation of the stretching rigidities, the shear rigidity, the in-plane Poisson's ratios, and the thermal expansion coefficients of the graphene from 300 K to 1000 K is shown in Table 3. It can be seen that the stretching rigidities are decreased slightly as temperature increases. However, the trend of the shear rigidity is opposite to that of the stretching rigidities, which is in agreement with the MD results of Lin et al. [7]. Like the single crystal copper, the NPR of the graphene sheet is increased when temperature rises. However, the thermal expansion coefficients of the graphene sheet are negative, which means that graphene contracts when temperature increases. The effect of different loading directions on NPR has been investigated in our previous work [45] where the changes of NPRs with an increased load in the zigzag and armchair directions were also studied. We found that the gap between the NPRs in the zigzag and armchair directions is remarkable when the load is small but the gap is gradually reduced with the increase in the load. It was believed that the geometric configuration in different chiral directions plays a key role, because the distance between neighboring carbon atoms is increased once deformation of the graphene is occurred, which causes the reduction of the C-C bonds. With the increase in the interatomic distance, the effect of geometric configuration becomes weak, which results in the reduction of the gap between the NPRs in those two directions.

The bending behavior is then performed to determine the effective thickness of graphene. As mentioned above, the small force is set to be 10^−4^ nN and 1 million steps are taken for the balance of the whole system. The center deflection of graphene is calculated as the average value among transverse displacements of the nearest carbon atoms to the center point. In our study, the strain load of 10^−6^/fs is applied on the MD model and the averaged lattice parameters for the time step are converged to within 0.0001 nm. The strain load 10^−6^/fs with unit fs^−1^ means that we apply about 2 × 10^−5^ nm as an incremental displacement per femtosecond on the MD model of graphene. The equilibrium state depends on the difference of lattice parameters within a time step. If the difference is lower than 0.0001 nm, the whole model can be regarded to be in an equilibrium state. The graphene is an atomic-scale two-dimensional hexagonal lattice made by carbon atoms. In a dimensionally fixed graphene, the geometric center point must be in a hexagon cell. In our computation, the transverse displacements of the six carbon atoms of the hexagon cell in the center area of graphene are averaged to obtain the deflection of the graphene for each time point. Applying Equation (15), we obtain the effective thickness of the graphene being 0.159 nm with the deflection of 5.784 Å at room temperature when the equivalent applied pressure *q* is 3.75 × 10^−3^ GPa. Then, the density of the graphene can be obtained, which is 4717 kg/m^3^. Finally, the effective moduli of graphene can also be obtained based on Equations (3)–(6).  Table 4 presents the elastic moduli *E*_11_ and *E*_22_ as well as the shear modulus *G*_12_ for the graphene sheet.

### 3.3. Material Properties of Graphene/Cu Nanocomposites

In this section, five different graphene weight fractions for the graphene-reinforced copper composites will be taken into consideration. The calculation of the graphene weight fractions can be achieved based on the number of carbon and copper atoms and their respective atom weight. Then, the volume fractions can be directly obtained by [66]

(19) $$\begin{array}{c} V_{\text{G}}=\frac{w_{\text{G}}}{w_{\text{G}}+\left(\rho^{\text{G}}/\rho^{\text{C}}\right)-\left(\rho^{\text{G}}/\rho^{\text{C}}\right)w_{\text{G}}}, \end{array}$$

where *V* and *w* denote the volume fraction and the weight fraction, respectively. *ρ* is the mass density. The superscripts or subscripts G and C represent graphene and copper, respectively. For example, one of our MD models for composites contains 112064 atoms in all, of which 14720 atoms are carbon and the rest are copper atoms. Accordingly, the obtained graphene weight fraction is 2.8%. Taking 8960 kg/m^3^ as the mass density of copper, the volume fraction of graphene is then calculated to be 5%. The considered five weight fractions and their corresponding volume fractions are listed in Table 5.

**Figure 2:**
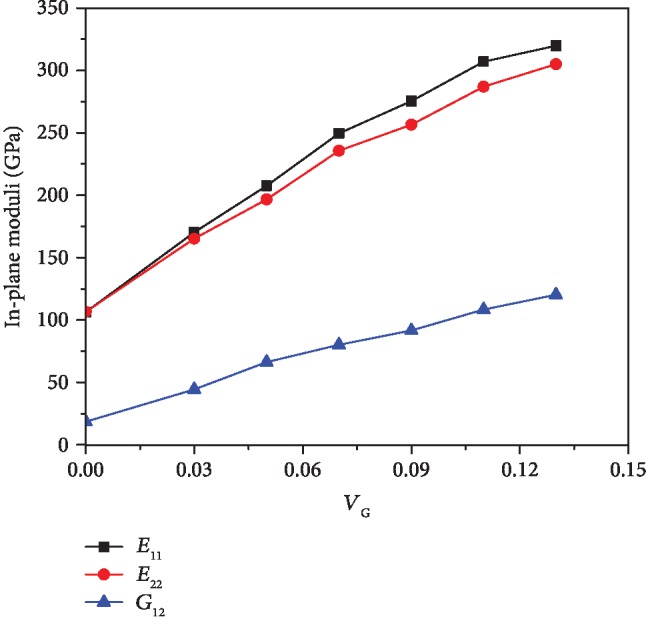
The variation of in-plane Young's moduli and shear modulus of composites with different graphene volume fractions at room temperature.

**Figure 3:**
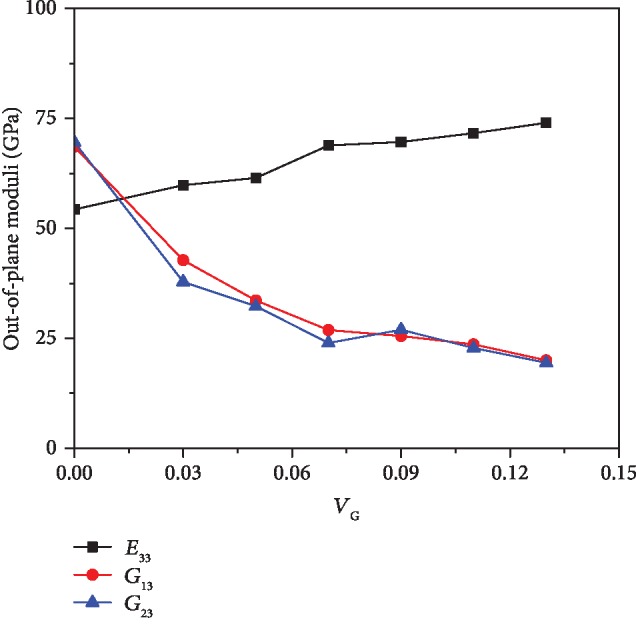
The variation of out-of-plane Young's modulus and shear moduli of composites with different graphene volume fractions at room temperature.

Figure 2 illustrates the effect of graphene volume fraction on the in-plane Young's moduli and shear modulus of the composites at room temperature (300 K). It is worth noting that the 11 direction is along the zig-zag direction of the graphene as well as the [1 1 0] crystal direction of the copper while the 22 direction is along the armchair direction of the graphene as well as the $\left[1\;\bar{1}\;0\right]$ crystal direction of the copper. We found that *E*_11_ is slightly larger than *E*_22_ and they are both improved as the volume fraction of graphene is increased. We also notice that the rising trend of the moduli becomes slower when the graphene volume is higher than 7%. The out-of-plane moduli, *E*_33_, *G*_13_, and *G*_23_, with different *V*_G_ are depicted in Figure 3. Although the Young's modulus in the thickness direction is enhanced by increasing the volume fraction of graphene, the incremental rate is relatively smaller when compared with that of the in-plane Young's moduli. It can be found that *E*_33_ is only improved by 7% with 13% volume fraction of graphene when compared with that of the pure copper. Unlike the in-plane shear modulus, the out-of-plane shear moduli, *G*_13_ and *G*_23_, are actually weakened by adding graphene reinforcement. We observe that the out-of-plane shear properties are degraded by as much as half when the composite contains only 5% volume fraction of graphene. The detailed moduli of the composites with different volume fractions of graphene under various temperatures are presented in Table 6.

**Figure 4:**
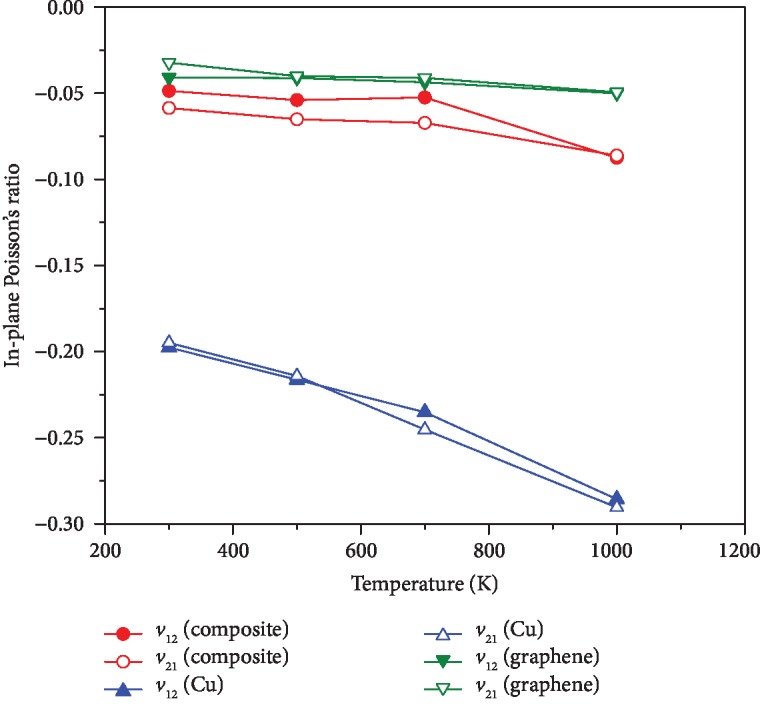
The effect of temperature on NPRs of the composite (*V*_G_ = 9%) and its component materials.

**Figure 5:**
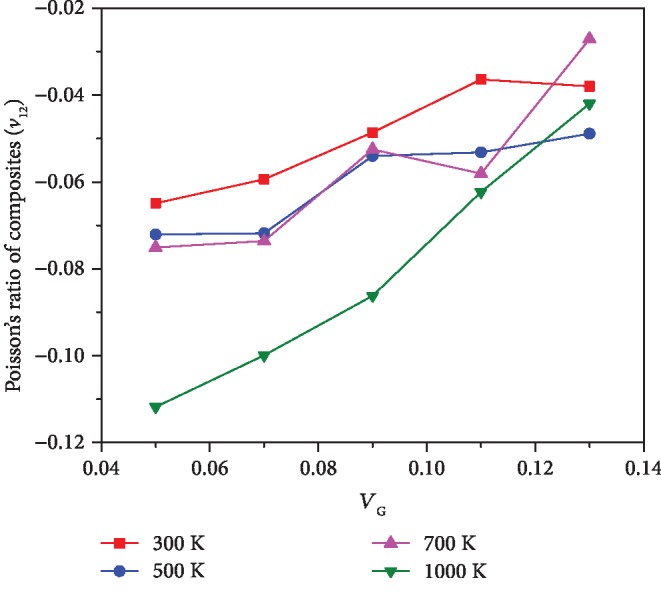
The effect of graphene volume fraction *V*_G_ on *ν*_12_ of the composite under different temperature conditions.

**Figure 6:**
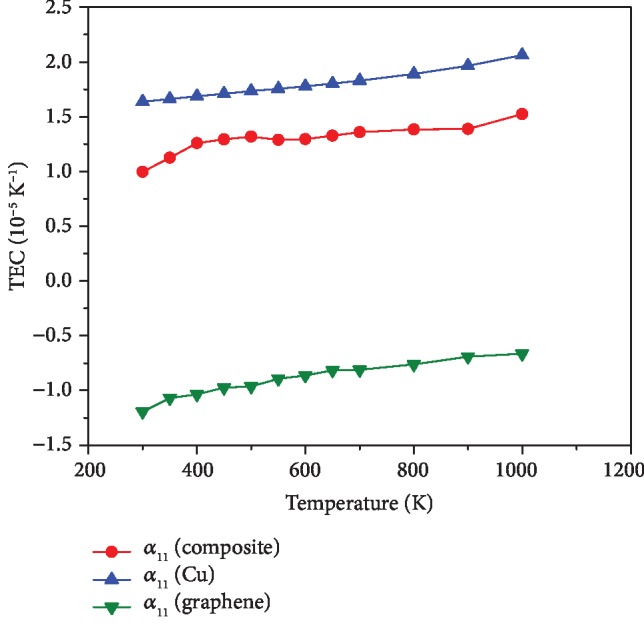
The effect of temperature on TEC of the composite (*V*_G_ = 9%) and its component materials.

In order to study the auxetic behavior of the graphene-reinforced copper composites, the NPRs of the composite with 9% graphene volume fraction and its component materials are all shown in Figure 4. As mentioned above, the graphene sheet and single crystal copper are both in-plane auxetic. Unlike in [21] where out-of-plane NPRs were obtained for graphene/Cu composites, in the current study, the in-plane NPRs of the resulting composite are obtained. From Figure 4, it can be found that the in-plane NPRs (*ν*_12_ and *ν*_21_) of the composite are between those of graphene and copper but closer to the graphene NPRs. With increase in temperature, the NPRs of the composite are decreased and the NPRs can reach about -0.09 at 1000 K. The effect of graphene volume fraction on *ν*_12_ of the composite is shown in Figure 5. Unlike the effect of temperature, the increase in graphene content may weaken auxetic characteristics of the composite. The TECs of the copper and graphene as well as the composite varying from 300 K to 1000 K are shown in Figure 6. Due to *α*_11_ and *α*_22_ for all three materials being very close, only their *α*_11_ is compared in Figure 6. Obviously, the TEC of graphene is negative but the TEC of copper is positive. The TEC of the composite is still positive but lower than that of the copper. In other words, the addition of graphene with negative TECs decreases the TECs of the copper based composite. Hence, internal stress may exist in the graphene-reinforced copper composites once temperature is varied. It is observed that the TEC trend of the composite is slightly different from that of graphene or copper due to the coupling effect.  Table 7 shows detailed NPRs and TECs of composites from 300 K to 1000 K. It is worth noting that the interaction between the carbon atom and copper atom is approximated by the L-J potential in the present MD models of graphene-reinforced composites. Hence, the influence of graphene reinforcement on the mechanical properties and Poison's ratio of the composites mainly depends on the mechanical loading transfer at the interface of the graphene sheet and copper matrix.

## 4. Conclusion

This study not only presents novel MD results for MMCs reinforced by graphene sheets but also provides a new approach for the design of auxetic materials and structures. The graphene sheet and single crystal copper both showing in-plane auxeticity are selected as reinforcements and matrix, and their detailed material properties are obtained through MD simulations. To determine the effective material properties of graphene-reinforced metal matrix composites (GRMMCs), the MD simulations are carried out under different graphene volume fractions and temperature ranges from 300 K to 1000 K. The results showed that the in-plane NPRs of GRMMCs are decreased as the temperature rises or the graphene volume fraction increases and the maximum NPRs may reach -0.11 at 1000 K when the volume fraction of graphene *V*_G_ is 5%.

## Figures and Tables

**Table 1:** In-plane material properties of single crystal copper under different temperatures (11 and 22 directions are, respectively, referred to as [1 1 0] and $\left[1\;\bar{1}\;0\right]$ crystal orientations).

Temperature (K)	*E*_11_ (GPa)	*E*_22_ (GPa)	*G*_12_ (GPa)	*ν*_12_	*ν*_21_	*α* (×10^−6^ K^−1^)
300	106.33	106.71	18.648	-0.1976	-0.1949	16.3766
500	95.576	96.224	16.684	-0.2164	-0.2142	17.3444
700	86.733	87.289	14.544	-0.2352	-0.2413	18.3063
1000	70.642	70.674	11.615	-0.2857	-0.2905	20.6236

**Table 2:** Out-of-plane material properties of single crystal copper under different temperatures (11 and 22 directions are, respectively, referred to as [1 1 0] and $\left[1\;\bar{1}\;0\right]$ crystal orientations).

Temperature (K)	*E*_33_ (GPa)	*G*_13_ (GPa)	*G*_23_ (GPa)
300	54.306	68.526	69.585
500	49.792	63.267	63.553
700	43.934	59.295	59.722
1000	39.966	50.74	50.923

**Table 3:** In-plane properties of monolayer graphene under different temperatures (11 and 22 directions are, respectively, referred to as zig-zag and armchair directions).

Temperature (K)	*C*_11_ (GPa nm)	*C*_22_ (GPa nm)	*C*_66_ (GPa nm)	*ν*_12_	*ν*_21_	*α*_11_ (×10^−6^ K^−1^)	*α*_22_ (×10^−6^ K^−1^)
300	357.27	339.82	128.91	-0.0409	-0.0392	-11.952	-11.948
500	353.24	330.04	131.71	-0.0412	-0.0389	-9.6269	-9.6267
700	349.71	327.29	137.62	-0.0436	-0.0411	-8.1185	-8.1177
1000	321.54	305.84	143.73	-0.0501	-0.0482	-6.6417	-6.6397

**Table 4:** Moduli of monolayer graphene under different temperatures (11 and 22 directions are, respectively, referred to as zig-zag and armchair directions).

Temperature (K)	*E*_11_ (TPa)	*E*_22_ (TPa)	*G*_12_ (TPa)
300	2.2470	2.1372	0.8108
500	2.2216	2.0757	0.8284
700	2.1994	2.0584	0.8655
1000	2.0223	1.9235	0.9040

**Table 5:** The weight fractions and their corresponding volume fractions (the mass densities of graphene and copper are 4717 kg/m^3^ and 8960 kg/m^3^, respectively).

Weight fraction	Volume fraction
2.8%	5%
3.7%	7%
4.8%	9%
6.1%	11%
7.1%	13%

**Table 6:** Temperature-dependent properties of graphene-reinforced copper composites (11 and 22 directions are, respectively, referred to as zig-zag and armchair directions, and the 33 direction is referred to as the thickness direction).

Temperature (K)	*V*_G_	*E*_11_ (GPa)	*E*_22_ (GPa)	*E*_33_ (GPa)	*G*_12_ (GPa)	*G*_13_ (GPa)	*G*_23_ (GPa)
300	5%	207.55	196.69	61.454	66.389	33.617	32.327
	7%	249.62	235.67	68.840	80.214	26.881	26.895
	9%	275.44	256.48	69.651	91.706	25.517	24.949
	11%	307.06	286.97	71.640	108.42	23.616	22.802
	13%	319.77	304.99	73.993	120.34	20.001	19.445

500	5%	193.15	183.94	55.773	62.092	31.536	31.009
	7%	235.50	221.18	61.819	77.036	25.845	25.809
	9%	258.90	243.71	68.271	87.200	24.257	23.893
	11%	293.37	273.66	69.588	100.92	21.853	20.786
	13%	296.39	288.72	72.029	112.29	19.283	17.660

700	5%	180.50	171.58	47.192	58.314	28.928	28.200
	7%	219.61	206.17	53.817	72.686	24.115	23.587
	9%	242.91	229.60	62.517	82.040	23.627	22.989
	11%	271.55	254.96	67.880	97.670	17.003	16.290
	13%	284.59	275.48	69.542	110.79	18.057	17.396

1000	5%	155.35	148.86	40.818	53.053	24.109	23.701
	7%	197.96	178.74	49.213	65.355	22.997	22.394
	9%	220.43	199.26	60.216	76.439	21.923	21.839
	11%	248.34	210.12	64.923	93.537	16.211	15.926
	13%	253.87	223.40	66.792	99.681	16.731	15.988

**Table 7:** Temperature-dependent NPRs and TECs of graphene-reinforced copper composites (11 and 22 directions are, respectively, referred to as zig-zag and armchair directions, and the 33 direction is referred to as the thickness direction).

Temperature (K)	*V*_G_	*ν*_12_	*ν*_13_	*ν*_23_	*α*_11_ (×10^−6^ K^−1^)	*α*_22_ (×10^−6^ K^−1^)
300	5%	-0.0649	0.6297	0.6512	1.4224	1.4194
	7%	-0.0594	0.5631	0.6034	1.0812	1.0813
	9%	-0.0486	0.5111	0.5088	0.9976	1.0021
	11%	-0.0364	0.4235	0.4598	0.8204	0.8204
	13%	-0.0380	0.4026	0.4348	0.7984	0.8014

500	5%	-0.0721	0.6298	0.6617	1.5037	1.5006
	7%	-0.0718	0.5726	0.6358	1.3848	1.3957
	9%	-0.0540	0.5494	0.5584	1.3174	1.3046
	11%	-0.0532	0.5345	0.5019	1.2199	1.2053
	13%	-0.0489	0.5025	0.4951	1.0232	1.0288

700	5%	-0.0751	0.6826	0.6742	1.6222	1.6278
	7%	-0.0736	0.6452	0.6294	1.4324	1.4286
	9%	-0.0525	0.5828	0.5763	1.3589	1.3489
	11%	-0.0581	0.5643	0.5011	1.2069	1.2066
	13%	-0.0271	0.5576	0.4317	1.1367	1.1422

1000	5%	-0.1118	0.6978	0.7063	1.7022	1.7331
	7%	-0.0994	0.6572	0.6453	1.7205	1.6899
	9%	-0.0875	0.6033	0.5791	1.5242	1.5416
	11%	-0.0676	0.5750	0.5854	1.3028	1.3016
	13%	-0.0415	0.5633	0.5955	1.3256	1.3083

## Appendix

A graphene sheet with length *a*, width *b*, and effective thickness *h*_e_ under a distributed transverse pressure *q* is considered. When the effective thickness is thin enough, the classical thin plate theory is adapted and the displacement field of an arbitrary point (*X*, *Y*) on the graphene can be expressed as

(A.1) $$\begin{array}{c} U_{1}=\bar{U}\left(X,Y,t\right)-Z\frac{\partial \bar{W}\left(X,Y,t\right)}{\partial X},\\ U_{2}=\bar{V}\left(X,Y,t\right)-Z\frac{\partial \bar{W}\left(X,Y,t\right)}{\partial Y},\\ U_{3}=\bar{W}\left(X,Y,t\right), \end{array}$$

where $\bar{U}$, $\bar{V}$, and $\bar{W}$ are midplane displacements in a Cartesian coordinate (*X*, *Y*, and *Z*) and *t* is the time. Then, the corresponding strains in the von Kármán type can be written as

(A.2) $$\begin{array}{c} \varepsilon_{1}=\frac{\partial \bar{U}}{\partial X}+\frac{1}{2}{\left(\frac{\partial \bar{W}}{\partial X}\right)}^{2}-Z\frac{\partial^{2}\bar{W}}{\partial X^{2}},\\ \varepsilon_{2}=\frac{\partial \bar{V}}{\partial Y}+\frac{1}{2}{\left(\frac{\partial \bar{W}}{\partial Y}\right)}^{2}-Z\frac{\partial^{2}\bar{W}}{\partial Y^{2}},\\ \varepsilon_{3}=\varepsilon_{4}=\varepsilon_{5}=0,\\ \varepsilon_{6}=\frac{\partial \bar{U}}{\partial Y}+\frac{\partial \bar{V}}{\partial X}+\frac{\partial^{2}\bar{W}}{\partial X\partial Y}-2Z\frac{\partial \bar{W}}{\partial X\partial Y}. \end{array}$$

The stress-strain relationship in a plane stress state can be expressed as

(A.3) $$\begin{array}{c} \left[\begin{array}{c} \sigma_{1}\\ \sigma_{2}\\ \sigma_{6} \end{array}\right]=\left[\begin{array}{ccc} Q_{11} & Q_{12} & 0\\ Q_{21} & Q_{22} & 0\\ 0 & 0 & Q_{66} \end{array}\right]\left[\begin{array}{c} \varepsilon_{1}\\ \varepsilon_{2}\\ \varepsilon_{6} \end{array}\right], \end{array}$$

where

(A.4) $$\begin{array}{c} \left[\begin{array}{c} Q_{11}\\ Q_{12}\\ Q_{22}\\ Q_{66} \end{array}\right]=\left[\begin{array}{cccc} \frac{1}{1-v_{12}v_{21}} & 0 & 0 & 0\\ 0 & \frac{v_{21}}{1-v_{12}v_{21}} & 0 & 0\\ 0 & 0 & \frac{1}{1-v_{12}v_{21}} & 0\\ 0 & 0 & 0 & 1 \end{array}\right]\left[\begin{array}{c} E_{11}\\ E_{11}\\ E_{22}\\ G_{12} \end{array}\right]. \end{array}$$

By employing the Hamilton principle, we have

(A.5) $$\begin{array}{c} \int_{t_{1}}^{t_{2}}\left(\delta U+\delta V-\delta K\right)dt=0, \end{array}$$

where

(A.6) $$\begin{array}{c} \delta U=\int_{\Omega }\int_{-h/2}^{h/2}\left(\sigma_{i}\varepsilon_{i}\right)dZdXdY\;\left(i=1,2,6\right),\\ \delta V=-\int_{\Omega }q\delta U_{3}dXdY,\\ \delta K=\int_{\Omega }\int_{-h/2}^{h/2}\rho \left[\frac{\partial U_{j}}{\partial t}\frac{\partial \left(\delta U_{j}\right)}{\partial t}\right]dZdXdY\;\left(j=1,2,6\right), \end{array}$$

where *Ω* is the area of the plate. Using the variational principle, the von Kármán-type nonlinear equations for the thin plate can be derived as [57]

(A.7) $$\begin{array}{c} L_{1}\left(\bar{W}\right)+L_{3}\left(\bar{F}\right)=\tilde{L}\left(\bar{W},\bar{F}\right)+q, \end{array}$$

(A.8) $$\begin{array}{c} L_{2}\left(\bar{F}\right)-L_{3}\left(\bar{W}\right)=-\frac{1}{2}\tilde{L}\left(\bar{W},\bar{W}\right), \end{array}$$

where $\bar{F}$ is the stress function defined by ${\bar{N}}_{x}=\partial^{2}\bar{F}/\partial Y^{2}$, ${\bar{N}}_{y}=\partial^{2}\bar{F}/\partial X^{2}$, and ${\bar{N}}_{xy}=-\partial^{2}\bar{F}/\partial X\partial Y$ and the linear operators are defined by

(A.9) $$\begin{array}{c} L_{1}\left(\;\right)=D_{11}^{*}\frac{\partial^{4}}{\partial X^{4}}+2\left(D_{12}^{*}+2D_{66}^{*}\right)\frac{\partial^{4}}{\partial X^{2}\partial Y^{2}}+2D_{22}^{*}\frac{\partial^{4}}{\partial Y^{4}}, \end{array}$$

(A.10) $$\begin{array}{c} L_{2}\left(\;\right)=A_{22}^{*}\frac{\partial^{4}}{\partial X^{4}}+\left(2A_{12}^{*}+A_{66}^{*}\right)\frac{\partial^{4}}{\partial X^{2}\partial Y^{2}}+A_{11}^{*}\frac{\partial^{4}}{\partial Y^{4}}, \end{array}$$

(A.11) $$\begin{array}{c} L_{3}\left(\;\right)=\left(2B_{26}^{*}-B_{61}^{*}\right)\frac{\partial^{4}}{\partial X^{3}\partial Y}+2\left(B_{16}^{*}-B_{62}^{*}\right)\frac{\partial^{4}}{\partial X\partial Y^{3}}, \end{array}$$

and *L*( ) contains the geometric nonlinearity terms in the von Kármán sense and can be given as

(A.12) $$\begin{array}{c} L\left(\;\right)=\frac{\partial^{2}}{\partial X^{2}}\frac{\partial^{2}}{\partial Y^{2}}-2\frac{\partial^{2}}{\partial X\partial Y}\frac{\partial^{2}}{\partial X\partial Y}+\frac{\partial^{2}}{\partial Y^{2}}\frac{\partial^{2}}{\partial X^{2}}, \end{array}$$

in Equations (A.9)–(A.11); [*A*_*ij*_], [*B*_*ij*_], and [*D*_*ij*_] (*i*, *j* = 1, 2, 6) are reduced stiffness matrices which are defined as

(A.13) $$\begin{array}{c} A^{*}=A^{-1},\\ B^{*}=-A^{-1}B,\\ D^{*}=D-{BA}^{-1}B, \end{array}$$

where *A*_*ij*_, *B*_*ij*_, and *D*_*ij*_ are the stiffness coefficients defined by

(A.14) $$\begin{array}{c} \left(A_{ij},B_{ij},D_{ij}\right)=\int_{-h/2}^{h/2}Q_{ij}\left(1,Z,Z\right)dZ. \end{array}$$

The solutions of Equations (A.7) and (A.8) can be expanded in the perturbation forms as

(A.15) $$\begin{array}{c} W=\underset{j=1}{\sum }\varepsilon^{j}w_{j}\left(x,y\right), \end{array}$$

(A.16) $$\begin{array}{c} F=\underset{j=0}{\sum }\varepsilon^{j}f_{j}\left(x,y\right), \end{array}$$

(A.17) $$\begin{array}{c} q=\underset{j=1}{\sum }\varepsilon^{j}\lambda_{j}. \end{array}$$

By substituting Equations (A.15)–(A.17) into Equations (A.7) and (A.8) and using a two-step perturbation approach [67], the solution of load can be written as

(A.18) $$\begin{array}{c} \frac{{qL}_{x}^{4}}{D_{11}h_{\text{e}}}=A_{W}^{\left(1\right)}\left(\frac{\bar{W}}{h_{\text{e}}}\right)+A_{W}^{\left(3\right)}{\left(\frac{\bar{W}}{h_{\text{e}}}\right)}^{3}+\cdots . \end{array}$$

Equation (A.18) is the load-deflection relationship that we used in this study to obtain the effective thickness of the graphene. Substituting *D*_11_ = *C*_11_*h*_e_^2^/[12(1 − *ν*_12_^*G*^*ν*_21_^*G*^)] into Equation (A.18) and multiplying both sides of Equation (A.18) by *h*_e_^3^, one has

(A.19) $$\begin{array}{c} A_{W}^{\left(1\right)}\bar{W}h_{\text{e}}^{2}=\frac{12\left(1-\nu_{12}^{\text{G}}\nu_{21}^{\text{G}}\right){qL}_{x}^{4}}{C_{11}}-A_{W}^{\left(3\right)}{\bar{W}}^{3}. \end{array}$$

From Equation (A.19), the final expression of *h*_e_ can be obtained as

(A.20) $$\begin{array}{c} h_{\text{e}}={\left[\frac{\left(12\left(1-\nu_{12}^{\text{G}}\nu_{21}^{\text{G}}\right){qL}_{x}^{4}/C_{11}\right)-A_{W}^{\left(3\right)}{\bar{W}}^{3}}{A_{W}^{\left(1\right)}\bar{W}}\right]}^{1/2}. \end{array}$$

## References

[1] Reddy C. D., Rajendran S., Liew K. M. (2006). Equilibrium configuration and continuum elastic properties of finite sized graphene. *Nanotechnology*.

[2] Sun T., Li Z.-J., Zhang X.-B. (2018). Achieving of high density/utilization of active groups via synergic integration of C=N and C=O bonds for ultra-stable and high-rate lithium-ion batteries. *Research*.

[3] Geim A. K., Novoselov K. S. (2007). The rise of graphene. *Nature Materials*.

[4] Lee C., Wei X., Kysar J. W., Hone J. (2008). Measurement of the elastic properties and intrinsic strength of monolayer graphene. *Science*.

[5] Huang X., Yin Z., Wu S. (2011). Graphene-based materials: synthesis, characterization, properties, and applications. *Small*.

[6] Wu P., Fang Z., Zhang A. (2019). Chemically binding scaffolded anodes with 3D graphene architectures realizing fast and stable lithium storage. *Research*.

[7] Lin F., Xiang Y., Shen H.-S. (2017). Temperature dependent mechanical properties of graphene reinforced polymer nanocomposites - A molecular dynamics simulation. *Composites Part B: Engineering*.

[8] Han S., Meng Q., Araby S., Liu T., Demiral M. (2019). Mechanical and electrical properties of graphene and carbon nanotube reinforced epoxy adhesives: experimental and numerical analysis. *Composites Part A: Applied Science and Manufacturing*.

[9] Wu G., Yu Z., Jiang L. (2019). A novel method for preparing graphene nanosheets/Al composites by accumulative extrusion-bonding process. *Carbon*.

[10] Hu Z., Tong G., Lin D. (2016). Graphene-reinforced metal matrix nanocomposites–a review. *Materials Science and Technology*.

[11] Dixit S., Mahata A., Mahapatra D. R., Kailas S. V., Chattopadhyay K. (2018). Multi-layer graphene reinforced aluminum - Manufacturing of high strength composite by friction stir alloying. *Composites Part B: Engineering*.

[12] Jagannadham K. (2012). Thermal conductivity of copper-graphene composite films synthesized by electrochemical deposition with exfoliated graphene platelets. *Metallurgical and Materials Transactions B*.

[13] Hwang J., Yoon T., Jin S. H. (2013). Enhanced mechanical properties of graphene/copper nanocomposites using a molecular-level mixing process. *Advanced Materials*.

[14] Zhang Y., Heim F. M., Bartlett J. L., Song N., Isheim D., Li X. (2019). Bioinspired, graphene-enabled Ni composites with high strength and toughness. *Science Advances*.

[15] Naseer A., Ahmad F., Aslam M. (2019). A review of processing techniques for graphene-reinforced metal matrix composites. *Materials and Manufacturing Processes*.

[16] Mokhalingam A., Kumar D., Srivastava A. (2017). Mechanical behaviour of graphene reinforced aluminum nano composites. *Materials Today: Proceedings*.

[17] Rezaei R. (2018). Tensile mechanical characteristics and deformation mechanism of metal-graphene nanolayered composites. *Computational Materials Science*.

[18] Montazeri A., Mobarghei A. (2018). Nanotribological behavior analysis of graphene/metal nanocomposites via MD simulations: new concepts and underlying mechanisms. *Journal of Physics and Chemistry of Solids*.

[19] Weng S., Ning H., Fu T. (2018). Molecular dynamics study of strengthening mechanism of nanolaminated graphene/Cu composites under compression. *Scientific Reports*.

[20] Sharma S., Kumar P., Chandra R. (2017). Mechanical and thermal properties of graphene–carbon nanotube-reinforced metal matrix composites: a molecular dynamics study. *Journal of Composite Materials*.

[21] Zhang C., Lu C., Pei L., Li J., Wang R., Tieu K. (2019). The negative Poisson's ratio and strengthening mechanism of nanolayered graphene/Cu composites. *Carbon*.

[22] Evans K. E., Nkansah M. A., Hutchinson I. J., Rogers S. C. (1991). Molecular network design. *Nature*.

[23] Fíla T., Zlámal P., Jiroušek O. (2017). Impact testing of polymer-filled auxetics using split Hopkinson pressure bar. *Advanced Engineering Materials*.

[24] Duc N. D., Seung-Eock K., Tuan N. D., Tran P., Khoa N. D. (2017). New approach to study nonlinear dynamic response and vibration of sandwich composite cylindrical panels with auxetic honeycomb core layer. *Aerospace Science and Technology*.

[25] Karathanasopoulos N., Reda H., Ganghoffer J. (2017). Designing two-dimensional metamaterials of controlled static and dynamic properties. *Computational Materials Science*.

[26] Imbalzano G., Linforth S., Ngo T. D., Lee P. V. S., Tran P. (2018). Blast resistance of auxetic and honeycomb sandwich panels: comparisons and parametric designs. *Composite Structures*.

[27] Jiang L., Hu H. (2017). Finite element modeling of multilayer orthogonal auxetic composites under low-velocity impact. *Materials*.

[28] Alderson K. L., Pickles A. P., Neale P. J., Evans K. E. (1994). Auxetic polyethylene: the effect of a negative Poisson's ratio on hardness. *Acta Metallurgica et Materialia*.

[29] Choi J. B., Lakes R. S. (1996). Fracture toughness of re-entrant foam materials with a negative Poisson's ratio: experiment and analysis. *International Journal of Fracture*.

[30] Howell B., Prendergast P., Hansen L. (1994). Examination of acoustic behavior of negative Poisson's ratio materials. *Applied Acoustics*.

[31] Huang C., Chen L. (2016). Negative Poisson's ratio in modern functional materials. *Advanced Materials*.

[32] Saxena K. K., Das R., Calius E. P. (2016). Three decades of auxetics research − materials with negative Poisson's ratio: a review. *Advanced Engineering Materials*.

[33] Caddock B. D., Evans K. E. (1989). Microporous materials with negative Poisson's ratios. I. Microstructure and mechanical properties. *Journal of Physics D: Applied Physics*.

[34] Herakovich C. T. (1984). Composite laminates with negative through-the-thickness Poisson's ratios. *Journal of Composite Materials*.

[35] Li C., Shen H.-S., Wang H. (2019). Nonlinear bending of sandwich beams with functionally graded negative Poisson's ratio honeycomb core. *Composite Structures*.

[36] Li C., Shen H.-S., Wang H. (2019). Nonlinear vibration of sandwich beams with functionally graded negative Poisson's ratio honeycomb core. *International Journal of Structural Stability and Dynamics*.

[37] Li C., Shen H.-S., Wang H. (2019). Thermal post-buckling of sandwich beams with functionally graded negative Poisson's ratio honeycomb core. *International Journal of Mechanical Sciences*.

[38] Nguyen T. D., Duc N. D. (2016). Evaluation of elastic properties and thermal expansion coefficient of composites reinforced by randomly distributed spherical particles with negative Poisson's ratio. *Composite Structures*.

[39] Gao Z., Dong X., Li N., Ren J. (2017). Novel two-dimensional silicon dioxide with in-plane negative Poisson's ratio. *Nano Letters*.

[40] Alderson A., Alderson K. L. (2007). Auxetic materials. *Proceedings of the Institution of Mechanical Engineers, Part G: Journal of Aerospace Engineering*.

[41] Lakes R. (1993). Advances in negative Poisson's ratio materials. *Advanced Materials*.

[42] Milstein F., Huang K. (1979). Existence of a negative Poisson ratio in FCC crystals. *Physical Review B*.

[43] Zakharchenko K. V., Katsnelson M. I., Fasolino A. (2009). Finite temperature lattice properties of graphene beyond the quasiharmonic approximation. *Physical Review Letters*.

[44] Jiang J.-W., Chang T., Guo X., Park H. S. (2016). Intrinsic negative Poisson’s ratio for single-layer graphene. *Nano Letters*.

[45] Fan Y., Xiang Y., Shen H.-S. (2019). Temperature-dependent negative Poisson’s ratio of monolayer graphene: prediction from molecular dynamics simulations. *Nanotechnology Reviews*.

[46] Scarpa F., Adhikari S., Phani A. S. (2009). Effective elastic mechanical properties of single layer graphene sheets. *Nanotechnology*.

[47] Qin Z., Qin G., Hu M. (2018). Origin of anisotropic negative Poisson’s ratio in graphene. *Nanoscale*.

[48] Korobeynikov S. N., Alyokhin V. V., Babichev A. V. (2018). On the molecular mechanics of single layer graphene sheets. *International Journal of Engineering Science*.

[49] Phliptom S. (1995). Fast parallel algorithms for short-range molecular dynamics. *Journal of Computional Physics*.

[50] Stukowski A. (2009). Visualization and analysis of atomistic simulation data with OVITO - the Open Visualization Tool. *Modelling and Simulation in Materials Science and Engineering*.

[51] Lebedeva I. V., Minkin A. S., Popov A. M., Knizhnik A. A. (2019). Elastic constants of graphene: comparison of empirical potentials and DFT calculations. *Physica E: Low-dimensional Systems and Nanostructures*.

[52] Tersoff J. (1988). Empirical interatomic potential for carbon, with applications to amorphous carbon. *Physical Review Letters*.

[53] Brenner D. W. (1990). Empirical potential for hydrocarbons for use in simulating the chemical vapor deposition of diamond films. *Physical Review B*.

[54] Erhart P., Albe K. (2005). Analytical potential for atomistic simulations of silicon, carbon, and silicon carbide. *Physical Review B*.

[55] Foiles S. M., Baskes M. I., Daw M. S. (1986). Embedded-atom-method functions for the fcc metals Cu, Ag, Au, Ni, Pd, Pt, and their alloys. *Physical Review B*.

[56] Daw M. S., Baskes M. I. (1984). Embedded-atom method: derivation and application to impurities, surfaces, and other defects in metals. *Physical Review B*.

[57] Shen H.-S. (2000). Nonlinear analysis of composite laminated thin plates subjected to lateral pressure and thermal loading and resting on elastic foundations. *Composite Structures*.

[58] Islam M. Z., Mahboob M., Lowe R. L., Bechtel E. S. (2013). Characterization of the thermal expansion properties of graphene using molecular dynamics simulations. *Journal of Physics D: Applied Physics*.

[59] Karadeniz Z. H., Kumlutas D. (2007). A numerical study on the coefficients of thermal expansion of fiber reinforced composite materials. *Composite Structures*.

[60] Chang S.-W., Nair A. K., Buehler M. J. (2012). Geometry and temperature effects of the interfacial thermal conductance in copper– and nickel–graphene nanocomposites. *Journal of Physics: Condensed Matter*.

[61] Hong Y., Li L., Zeng X. C., Zhang J. (2015). Tuning thermal contact conductance at graphene-copper interface via surface nanoengineering. *Nanoscale*.

[62] Wang X., Wang X., Liu M., Crimp M. A., Wang Y., Qu Z. (2018). Anisotropic thermal expansion coefficient of multilayer graphene reinforced copper matrix composites. *Journal of Alloys and Compounds*.

[63] Zhang S., Xu Y., Liu X., Luo S. N. (2018). Competing roles of interfaces and matrix grain size in the deformation and failure of polycrystalline Cu–graphene nanolayered composites under shear loading. *Physical Chemistry Chemical Physics*.

[64] Montazeri A., Panahi B. (2018). MD-based estimates of enhanced load transfer in graphene/metal nanocomposites through Ni coating. *Applied Surface Science*.

[65] Huang S. P., Mainardi D. S., Balbuena P. B. (2003). Structure and dynamics of graphite-supported bimetallic nanoclusters. *Surface Science*.

[66] Rafiee M. A., Rafiee J., Wang Z., Song H., Yu Z. Z., Koratkar N. (2009). Enhanced mechanical properties of nanocomposites at low graphene content. *ACS Nano*.

[67] Shen H.-S. (2013). *A Two-Step Perturbation Method in Nonlinear Analysis of Beams*.

